# Anesthesia Providers are Obligated to Give Patients the Alternatives to General Anesthesia when Obtaining Informed Consent

**Published:** 2014

**Authors:** Michael R Pascarella, Jason D Walls, Renyu Liu, Linda Chen

**Affiliations:** Department of Anesthesiology and Critical Care, Perelman School of Medicine at the University of Pennsylvania

Informed consent is a critical part of the pre-anesthetic interview and physician-patient relationship. Informed consent should include the nature of the anesthetic plan, the material risks and benefits, as well as the alternatives to the plan. A recent study indicated that even though systematic informed consent is required and performed by providers, patient comprehension is still lacking.^1^ Furthermore adequate training in the principles of informed consent is needed in the early stages of residency training.^2^ In this editorial, we will discuss an important legal case involving an anesthesiologist in order to discuss the legal requirements of informed consent, strategies to improve the physician-patient relationship, and avoid litigation

## Facts of the Case

The Plaintiff in this case, LB, arrived at the hospital in the afternoon for exploratory abdominal surgery to be performed the next day. Upon arrival LB spent 20 minutes signing several forms, including one that indicated he was aware of the risks of receiving general anesthesia for his surgical procedure. After LB completed the preanesthetic evaluation “checklist”, he met with Dr. D, the defendant, for a preoperative evaluation. LB testified that during his meeting Dr. D told him that he recommended general anesthesia, that he would personally perform the procedure, and asked if he had any questions about the proposed anesthetic plan. LB also testified that Dr. D did not disclose any of the risks associated with general anesthesia or any of the available alternative types of anesthesia.

The following morning LB was brought to the operating room and was met by the co-defendant, SK, a nurse anesthetist, who explained that she would be performing the anesthesia under the supervision of Dr. D. LB voiced no complaints about her performing the anesthesia and did not note the absence of Dr. D.

Soon after SK began to administer medications for the induction of general anesthesia, LB’s airway became partially blocked and he began having difficulty breathing. SK attempted corrective measures, but they were unsuccessful, and she then called for help. The exact time that elapsed between when SK first recognized the critical situation and when she first called Dr D. for help was a contested issue at the trial. Within minutes of the call for help numerous doctors entered the room including Dr. D. After several attempts, the doctors were unsuccessful in establishing an airway for LB and soon thereafter the patient went into cardiac arrest.

The plaintiffs presented evidence that as a result of LB’s cardiac arrest he suffered severe physical and mental impairments. Plaintiffs filed an action against Dr. D and SK alleging: 1) the violation of the informed consent doctrine; 2) negligence by Dr. D in his preoperative evaluation of LB and allowing SK to administer the anesthesia in the absence of Dr. D; and 3) negligence by SK in the administration of the general anesthetic medications, the attempts at resuscitating LB once the problem realized, and the delay in calling Dr. D for help.

## Legal Analysis

The trial court initially dismissed LB’s claim for lack of informed consent, but the Superior Court reversed the decision. The Superior Court found that LB had made a prima facie case for lack of informed consent and the case was remanded for a new trial. (Brown v. Dahl, 41 Wn. App. 565; 705 P.2d 781; 1985 Wash. App. LEXIS 2860).^3^

Under the state malpractice law where the case was held, a health care provider must provide the patient with the nature of the proposed treatment as well as the benefits, material risks, and alternatives, including nontreatment. A fact is determined to be material if a “reasonably prudent person, in the position of the patient, would attach significance to it deciding whether or not to submit to the proposed treatment”. (RCW 7.70.050(2))

LB presented expert testimony to show that blocked airways, respiratory compromise, cardiac arrest, and death were all risks associated with general anesthesia. The expert also testified that there are various alternatives to general anesthesia, including local, regional, or no anesthesia. Furthermore the expert testified that Dr. D did not conduct an adequate preoperative consultation with LB to determine his candidacy for general anesthesia and violated the customary standard in obtaining informed consent by failing to inform LB of the risks and alternatives to general anesthesia. Dr. D admitted at trial that he did not want to frighten LB with facts relating to risks and availability of alternatives.

The Superior Courts reasoning was based on a patient-based “objective” rather than a “subjective” standard. Consequently, the legal test was not whether LB himself (subjective) would have chosen a different alternative to general anesthesia but whether a reasonably prudent person (patient-based standard) in LB’s position (objective) would have chosen a different form of anesthesia if he had known of the alternatives and risks.

In the trial court Dr. D’s expert made an argument that the non-disclosed alternative forms of anesthesia (regional, local, or no anesthesia) were not any safer, and may even be more dangerous than general anesthesia. However, the Superior Court disagreed with this reasoning because evidence that a reasonably prudent patient in LB’s position would have chosen another form of anesthesia than general anesthesia. Furthermore they found that Dr. D violated the customary practice in obtaining informed consent by failing to give the risks and alternatives.

The Superior Court reasoned that even though the alternative forms of anesthesia such as regional or local had inherent risks themselves, (which may have even been greater than general anesthesia), LB should have been made aware of them and been given the ability to make the decision himself. Even though LB did not have any questions about the anesthesia, Dr. D is still obligated to go over the risks and alternatives because LB might have made a different choice. Once it was shown that Dr D. failed to inform the patient of a material fact (alternatives to general anesthesia in this case), and LB gave consent without being fully aware of these material facts, then the jury ultimately decided whether a reasonably prudent patient under similar circumstances would have consented if they had known about the alternatives to general anesthesia (RCW 7.70.050).

## Litigation Strategy

Today, informed consent is infrequently the key reason a plaintiff brings a claim. Informed consent is estimated to account for only 1% of claims brought against anesthesiologists.^4^ However, informed consent issues remain a very important secondary issue of litigation strategy that has the potential to add liability by increasing the likelihood that a plaintiff will bring a claim and the frequency of payout in medical negligence cases.^4^ Thus although consent was not the primary reason the anesthesiologist was sued, improper consent can call into question the physician’s compassion, professionalism, and diligence while distracting the jury from the primary issue of the case.^4^

Currently, both physician-based as well as patient-centered informed consent standards exist. The physician-based standard, effective in 23 states, requires the informed consent to include risks, benefits, and alternatives that a “reasonably prudent practitioner” would discuss.^5^ In this paternalistic standard the physician has the discretion to decide how much information to tell patients which clearly favors the physician. Alternatively, the patient-based standard informed consent must provide the risks, benefits, and alternatives that a reasonable patient would want to know.^5,6^ This standard is more objective and favors the patient by requiring the physician to disclose any material risks that a reasonable person in the patient’s position would want to know.

The deficiencies of both standards have led to the exploration of shared decision making.^5^ Shared medical decision making is a process that involves the physician discussing the risks and benefits of all treatment options, the physician’s professional advice, the patient’s personal preferences and expectations about the treatment plan, and finally a “mutual medical decision.”^5^ Shared medical decision making attempts to improve patient autonomy and understanding as well as increased communication between patient and physician.^5^

## Strategies to Avoid Litigation

Thorough documentation of the consent process is vital in avoiding litigation. First it is important for an anesthesiology practice to have its own specific anesthesia consent that is separate from the hospital and surgical consents. The anesthesia consent should detail the risks, benefits, and alternatives unique to anesthesia which can be very different from surgical risks. A one page consent is also preferred over a multi-page consent as it decreases the chance of losing the signature page and makes it harder for a patient to argue that he did not understand the form or had insufficient time to read it.^7^

A handwritten consent note is also not advisable nor optimal as it lacks enough detail to defend against a malpractice claim especially if the claim is filed years after the incident and the anesthesiologist is forced to rely on the record to recall the conversation regarding the consent.^7,8^

An anesthesia consent form signed by the patient by no means absolves the anesthesiologist from liability. A patient can argue that consent never actually took place despite signing the document, by arguing that they did not understand the written consent, that it was signed under duress or mental impairment by medications, or that specific risks and alternatives were not discussed. The consent document can also be viewed by juries as a generic hospital form that the patient does not understand but is required to sign.

It is also very important to document in a way that shows the consent was an interactive process with the patient. Underlining and circling words and handwriting on the consent form will also help the anesthesiologist defend themselves by showing that he or she specifically discussed certain risks, the patient was involved in the consent, and understands it.^7^

It is also advisable to discuss the alternatives to general anesthesia with the patient and to give recommendations using a risk benefit analysis. Most patients will likely end up agreeing with an anesthesiologist’s professional opinion.

Patients need to be told about the material risks of anesthesia, however many physicians are unclear about what constitutes “material”. Usually material risks are considered either common but minor injuries (such as tooth damage and nausea), or rare but severe injuries (such as death or nerve injury).

The most important defense is to have a detailed conversation about the anesthetic risks and alternatives with every patient. Even if the alternatives to general anesthesia are not feasible or preferred by the physician, it is still important to explain to patients the thought process and involve them in the conversation. Patients are more likely to sue a physician if they dislike them or feel that the physician was not compassionate.

In conclusion, it is important to remember that informed consent is not just a signed document, it is an interactive discussion between anesthesiologist and patient that exemplifies the principles of shared decision making.^9^ It is important for patients to understand the unpredictable nature of the perioperative course and be told about the potential for unexpected adverse events in addition to alternative forms of anesthesia.^4^

While informed consent issues are rarely the primary reason for a claim, it is a very important secondary issue in negligence cases that can be used in the plaintiff’s attorney’s arsenal to destroy the credibility of the anesthesiologist and cast doubt on the care that was provided by the anesthesiologist.^4^ While anesthesiologists can not eliminate the risk of litigation they can certainly decrease their liability by compassionate communication with patients and families, and documenting these interactions in a thorough and shared informed consent process.
